# Efficiency of red cell distribution width in identification of children aged 1-3 years with iron deficiency anemia against traditional hematological markers

**DOI:** 10.1186/1471-2431-14-8

**Published:** 2014-01-15

**Authors:** Sunil Sazawal, Usha Dhingra, Pratibha Dhingra, Arup Dutta, Hiba Shabir, Venugopal P Menon, Robert E Black

**Affiliations:** 1 Center for Micronutrient Research, Annamalai University, Annamalai Nagar, India; 2 Department of International Health, Johns Hopkins Bloomberg School of Public Health, 615, North Wolfe Street, Baltimore, MD 21205-2103, USA; 3 Center for Public Health Kinetics, New Delhi, India

**Keywords:** Iron deficiency anemia, Red cell distribution width, RDW, Receiver’s operating characteristic curve, ROC, Screening, Sensitivity, Specificity, Children

## Abstract

**Background:**

Current strategy to identify iron deficiency anemia relies on markers involving high costs. Reports have suggested red cell distribution width (RDW) as a potential screening test for identifying iron deficiency anemia (IDA) but studies in pediatric populations are lacking. Our study elucidates the discriminative ability of RDW for detecting IDA among young children.

**Methods:**

2091 blood reports of children aged 1–3 years from an urban low socio-economic population of Delhi were analyzed to evaluate the sensitivity of RDW in discriminating IDA using receiver’s operating characteristic curve. Hemoglobin and RDW were estimated using coulter, zinc protoporphyrin with AVIV fluorometer and serum ferritin by enzyme linked immunosorbent assay.

**Results:**

A total of 1026 samples were classified as iron deficient anemia using gold standard. As a marker of overall efficiency, area under the curve for RDW was 0.83 (95% CI, 0.81- 0.84; p < 0.001). Sensitivity of RDW at cut-off of 18% to detect iron deficiency anemia was 76.5% and specificity 73.1% yielding a positive predictive value of 73% and negative predictive value of 76%. At a cut-off of RDW 16.4%, the sensitivity was 94% and at a cut-off of 21%, the specificity was 95%. Combination of hemoglobin ≤10 g/dL and RDW >15%, yielded a sensitivity of 99% and specificity of 90%. These data suggest that simple coulter analysis estimating hemoglobin and RDW can be used for identification of children in need for iron therapy.

**Conclusions:**

In India and similar settings, RDW >15% with hemoglobin ≤10.0 g/dL identifies iron deficient anemic children without need for iron status markers which could help reduce cost of management especially in poor settings.

**Trial registration:**

Clinicaltrials.gov NCT00255385.

## Background

Iron deficiency is the most common micronutrient deficiency among Indian preschool children contributing to increased burden of morbidity and mortality and the most significant negative consequence of iron deficiency is iron deficiency anemia (IDA). Recent NFHS–III surveys (2005–06) have shown that 70-85% (approx. 79.2%) of Indian young children have anemia [1]. IDA is attributed to inadequate iron intake, poor bioavailability of iron or high nutritional needs during childhood which is further exacerbated by chronic intestinal blood losses due to helminth infections and in many areas due to severe malarial infections [2,3]. Studies have shown that iron deficiency causes delay in cognitive development and poor motor and sensory system functioning and that iron supplementation in early years may prevent these complications among children [4]. Conversely, there is an evidence suggesting that routine iron treatment in non-iron deficient children may have adverse consequences for morbidity and infections [5,6]. Therefore, it is very important to detect iron deficiency (ID) at its earliest stage in children especially in a low resource setting and replenish the iron stores by proper supplementation, thereby preventing many of the adverse developmental and behavioral effects caused by IDA. Currently, the detection of IDA is largely dependent upon quantification of biochemical markers like serum ferritin (SFr), serum transferrin (STr) and zinc protoporphyrin (ZnPP) which are not routinely available and affordable in developing countries due to high costs. Moreover, these tests are altered by inflammation, which limits their applicability for clinical interpretation, especially in areas with high infection rates. Another limitation of the commonly used hematological tests is their poor sensitivity or specificity as they can be modified by conditions other than iron deficiency. Studies have shown that RDW in addition to other hematological markers like mean corpuscular volume (MCV) and hemoglobin can be used as a differential diagnostic tool for identification of iron deficiency anemia [7-9]. Various studies also show that the onset of iron deficiency anemia can be predicted using automated blood analyzers [7], as a low haemoglobin level along with a high level of anisocytosis detectable by red cell distribution width prove to be good indicators of changes in blood due to depleted iron stores [8]. It seems that the earliest hematological manifestation of iron deficiency is marked by an elevated level of RDW [9] and reports have shown that it is a cost-effective screening tool for early diagnosis of IDA in comparison to SFr and ZnPP [9-11]. The red blood cell (RBC) distribution width, a measure of variations in the width of circulating RBCs, reported as a part of complete blood count [12] has been known to be of value in the discrimination of iron deficiency anemia from other causes of microcytic anemia, but studies in pediatric populations are lacking. Thus, in the present study we evaluated the discriminative ability of RDW diagnostic test for detecting iron deficiency anemia among children aged 1–3 yrs in a low socio-economic setting using receiver’s operating characteristic curve (ROC) analyses.

## Methods

These findings are from a community based double blind randomized controlled trial conducted in Sangam Vihar, a peri-urban population in New Delhi, India to evaluate the effects of fortified milk for one year on common childhood morbidities, hematological markers (anemia/iron stores), growth and development of young children aged 1–3 years. In this trial we evaluated the effect of 2 separate interventions in comparison to their respective controls. The findings of these studies have been published previously [13,14]. The study protocol was approved by the human research and ethical review boards of the Johns Hopkins Bloomberg School of Public Health, USA and the Annamalai University, India. Informed written consent was obtained from the parents of the children who were willing to participate in the study.

Between April 2002 and April 2003, all eligible consented children were scheduled to visit the clinic for the baseline evaluation. At the clinic, study physician carried out detailed physical examination of child and socio-economic/demographic information of the family was collected. Baseline and end study blood sample reports were analyzed and a total of 2091 samples were included in this analysis.

### Laboratory investigations

Approximately 3 ml of venous blood sample was collected using a trace element-free syringe and immediately transferred into ethylenediaminetetraacetic acid (EDTA) vials and trace element-free heparin vials. Plasma was separated within 15 minutes of blood collection, and the contents of aliquot were transferred into trace element-free Eppendorf plastic tubes for storage at -20°C. The EDTA blood was analyzed on the same day with Coulter automated flow cytometer (Beckman Coulter, Fullerton, CA) for a detailed hemogram. One drop of blood was used for estimating ZnPP using a hematofluorometer (Aviv Biomedical, Lakewood NJ, USA). The hematoflurometer was calibrated and quality control checks were routinely performed with controls and calibrators provided by the manufacturer (AVIV Biomedical, Lakewood, NJ, USA). SFr was estimated using a commercial enzyme linked immunosorbent assay (Ramco Laboratories, Houston, USA).

In retrospective design, we analyzed hematological parameters of children aged 1–3 years. Anemia was defined as hemoglobin (Hb) concentration ≤10 g/dL. A lower cut-off was selected instead of the World Health Organisation (WHO) cut-off of 11 g/dL because majority of the iron deficient anemic children had Hb ≤10 g/dL. In order to test the sensitivity and specificity of RDW, the gold standard definition used for categorizing iron deficient anemia was: Hb concentration ≤10 g/dL and SFr <11 μg/L or ZnPP >80 μmol/mole of heme [15].

ROC analysis was performed to examine the sensitivity and specificity of RDW in discriminating IDA. Positive and negative predictive values and area under the curve were also calculated. ROC curve analysis was obtained by plotting sensitivity versus 1-specificity. This method allows comparison of the sensitivity of a given test to that of another at the same level of specificity. The sensitivity and specificity along with positive and negative predictive value at various cut-offs of RDW was calculated against the gold standard definition for iron deficiency anemia to arrive at an optimal cut-off value in our population. After obtaining a cut-off value of RDW a simple algorithm was used where RDW (cut-off value) and Hb ≤10 g/dL were used as a predictor for classifying IDA.

All statistical analysis was carried out using SPSS/PC Statistical ProgramVersion 18.0 (SPSS, Chicago, IL) and STATA version 10.0 (StataCorp, College Station, TX).

## Results

Basic demographic and biochemical characteristics of samples with iron deficient anemia and without iron deficient anemia are shown in Table 1. Of the 2091 blood reports of children analyzed, 1026 samples (49.06%) were classified as iron deficient anemia by gold standard. There was a mark difference in the values for various biochemical markers in iron deficient anemic and non-iron deficient anemic children. Mean values of Hb, mean corpuscular hemoglobin (MCH), MCV and SFr were markedly higher in non iron deficient anemic children as compared to iron deficient anemic children. As many studies have found SFr [16,17] and ZnPP [18] as one of the best biochemical indicators of iron deficiency anemia hence we used Hb along with SFr or ZnPP to define IDA for the present analysis. 

**Table 1:** Demographic and biochemical profile of samples with iron deficient anemia and without iron deficient anemia

**Variables**	**Samples with iron deficiency anemia (n = 1026)**	**Samples without iron deficiency anemia (n = 1065)**
Mean age (months)	25.5 ± 8.4	31.6 ± 9.8
Gender: males (%)	522 (50.9)	553 (51.9)
Mean hemoglobin (g/dL)	8.4 ± 1.2	10.9 ± 1.0
Mean MCH (pg)	20.0 ± 3.3	24.2 ± 2.3
Mean MCV (fl)	69.6 ± 8.4	78.9 ± 6.4
Mean RDW (%)	19.9 ± 2.4	16.8 ± 2.5
Mean serum ferritin (μg/L)	6.2 ± 5.8	16.3 ± 14.1
Mean ZnPP (μmol/mole of heme)	229.7 ± 126.3	74.4 ± 45.6

Results are given as the mean ± SD unless specified.

*Abbreviations*: *MCH* Mean corpuscular hemoglobin, *MCV* Mean corpuscular volume, *RDW* Red cell distribution width, *ZnPP* Zinc protoporphyrin.

**Figure 1:**
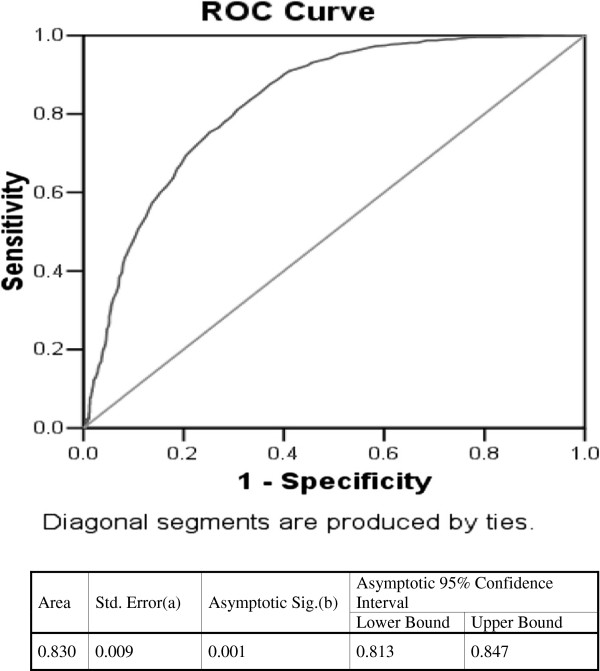
**ROC curve analysis.** Receiver operating characteristic curve analysis for RDW detecting iron deficiency anemia. The diagonal line represents the ROC curve for a test with no clinical value (i.e. area under the curve = 0.500).

ROC analysis of RDW for detecting iron deficiency anemia is shown in Figure 1. As a marker of overall efficiency, area under the curve for RDW was 83% (95% CI, 81% - 85%; p < 0.001) (Figure 1). Table 2 shows the sensitivity, specificity, positive and negative predictive values at various cut-offs of RDW against the gold standard definition for iron deficiency anemia. Sensitivity of RDW at cut-off of 18% to detect iron deficiency anemia was 76.5% and specificity of 73.1%. This cut-off yielded a positive predictive value of 73% and negative predictive value of 76%. At a cut-off of RDW 16.4%, the sensitivity was 94.2% and at a cut-off of 21%, the specificity was 95%. The algorithm using RDW value of >15% with Hb ≤10 g/dL was found to be more efficient. A second ROC analysis was performed using this algorithm as a predictor of IDA. Combination of Hb ≤10 g/dL and RDW >15%, yielded a sensitivity of 99% and specificity of 90%. The positive predictive value was 90% and the negative predictive value was around 99%.

**Table 2:** Sensitivity, specificity, positive and negative predictive values of RDW in diagnosing iron deficiency anemia

**Cut-offs (values in %)**	**Sensitivity (%)**	**Specificity (%)**	**PPV (%)**	**NPV (%)**
RDW- 18%	76.5	73.1	73.21	76.4
RDW- 16.4%	94.2	50.7	64.74	90.1
RDW- 21%	28.5	95	84.56	58.03
Hb ≤10 g/dL and RDW >15%	99	90	90.49	98.9

*Abbreviations*: *RDW* Red cell distribution width, *Hb* Hemoglobin, *PPV* Positive predictive value, *NPV* Negative predictive value.

## Discussion

The high incidence of IDA in children emphasizes the need for the cost effective and reliable tool in diagnosing IDA. A number of different indicators, such as hemoglobin, hematocrit, serum ferritin, transferrin saturation, erythropoietin, erythrocyte protoporphyrin, serum iron, mean corpuscular volume, mean corpuscular hemoglobin concentration have been used to evaluate IDA [11,19-21]. But the drawbacks of these tests are that many of them are expensive and require sophisticated laboratories, while others have been found to have a low specificity. It has been seen that anisocytosis occurs, where the erythrocytes produced are of smaller than average size and having a large size variation, due to inadequate iron supply. The morphology and function of erythrocytes at molecular level has been known to be disturbed due to iron deficiency anemia [22]. Therefore, an increase in RDW values may occur in IDA allowing an early detection of ID before reduction in MCV occurs. RDW has been reported to have a high predictive value for IDA [9,23] and can differentiate beta-thalassemia from other causes of anemia in populations [24,25]. Our results corroborate the view that RDW evaluated in a large sample performed very well as a screening diagnostic test for identifying iron deficiency anemia. These findings are similar to the findings of earlier studies conducted in other settings and support the usage of RDW as a screening tool for identifying iron deficiency anemia [26].

Other studies found the sensitivity of RDW to be very high (96 -100%) in detecting iron deficiency anemia [27,28]. On the contrary, there is a report of a limited specificity of RDW for diagnosis of IDA among children with microcytic hypochromic anemia [29]. At a cut-off value of 17.4%, as obtained from the ROC curve, the sensitivity and specificity of RDW in diagnosis of IDA were 81.0% and 53.4% and a positive and negative predictive value of 63.0% and 72.2%, respectively.

One of the other approaches used to predict IDA is the use of indexes such as Mentzler’s, discriminant function, Srivastava’s, Shine and Lal’s, MCV/MCH indices which are based on many hematological parameters instead of one [30]. In our study also, when we used Hb and RDW together the sensitivity and specificity improved considerably with high positive and negative predictive values. These data suggest that the combined approach of using Hb ≤10 g/dL and RDW >15% (sensitivity of 99% and specificity of 90%, positive predictive value of 90.5% and negative predictive value of 98.9%) performs well obviating the need for using expensive biochemical tests for diagnosing iron deficiency anemia in a low resource setting.

The strengths of the present study include the large number of standardized measurements and the use of ROC curves, which can summarize all the sensitivities and specificities in one diagram and can identify which cut-off/indicator has the highest sensitivity and specificity for the predictor variable. The prevalence of thalassaemia trait was 1.4% and thalassaemia major was 0% in the study population. Our results suggest very low prevalence of thalassaemia in our population and can thus be easily extrapolated in other similar settings.

The limitation of the present study is that a higher prevalence of subclinical infections, latent inflammatory disorders and other nutritional deficiencies like folic acid in our population, unlike the Western population, can falsely raise SFr levels, thereby suggesting that probably we need to redefine the acceptable normal range of SFr levels among our population. However, we have in our recent studies included the estimation of α1-Acid glycoprotein and C-reactive protein as markers for infections (Unpublished data; ClinicalTrials.gov Identifier: NCT00980421). The etiological fraction contributed by positivity of either or both to overall anemia prevalence was very low and correcting for it or after eliminating children with positive values did not change the prevalence estimates for anemia. In addition, although, the subjects of the study were from a randomized controlled trial for fortified milk, the results reported in this manuscript are retrospective observations. Retrospective studies are susceptible to bias in data selection and analysis. Furthermore, confounding variables may go unrecognized because of inadequate knowledge of how they interrelate with the outcome of interest thus rarely establishes the causal relationships.

## Conclusions

In conclusion, RDW > 15% and hemoglobin ≤10.0 g/dL measured using a simple coulter can be used as a valuable screening tool for identifying children with iron deficiency anemia in a low socio-economic setting. Although it needs to be further investigated in other populations, there is no reason to believe that results will vary from the present study. If these findings are confirmed in other settings as well, it offers a very useful tool for screening iron deficient anemic children without need for more expensive iron status marker investigations.

## Abbreviations

EDTA: Ethylenediamine tetraacetic acid; Hb: Hemoglobin; ID: Iron deficiency; IDA: Iron deficiency anemia; MCH: Mean corpuscular hemoglobin; MCV: Mean corpuscular volume; NFHS: National family health survey; RBC: Red blood cell; RDW: Red cell distribution width; ROC: Receiver’s operating characteristic curve; SFr: Serum ferritin; STr: Serum transferrin receptors; ZnPP: Zinc protoporphyrin; WHO: World Health Organisation.

## Competing interest

The authors declare that they have no competing interest.

## Authors’ contributions

SS, VM and RB coordinated the trial and made a primary contribution to its development, rationale, design, and undertaking, analysis of data, and revised the manuscript for important intellectual content. UD and AD contributed to implementation of the trial, quality control and were responsible for programming, data management, and analysis. PD contributed to the analysis of data and manuscript preparation. HS contributed to revising and analyzing the manuscript. All authors read and approved the final manuscript.

## Pre-publication history

The pre-publication history for this paper can be accessed here:

http://www.biomedcentral.com/1471-2431/14/8/prepub
